# Functions of Arp2/3 Complex in the Dynamics of Epithelial Tissues

**DOI:** 10.3389/fcell.2022.886288

**Published:** 2022-04-26

**Authors:** Emmanuel Martin, Magali Suzanne

**Affiliations:** ^1^ Molecular, Cellular and Developmental Biology (MCD), Centre de Biologie Intégrative (CBI), Université de Toulouse, CNRS, Toulouse, France; ^2^ FR3743 Centre de Biologie Intégrative (CBI), Toulouse, France

**Keywords:** ARP2/3 complex, branched actin, epithelial dynamics, morphogenesis, actin networks interplay

## Abstract

Epithelia are sheets of cells that communicate and coordinate their behavior in order to ensure their barrier function. Among the plethora of proteins involved in epithelial dynamics, actin nucleators play an essential role. The branched actin nucleation complex Arp2/3 has numerous functions, such as the regulation of cell-cell adhesion, intracellular trafficking, the formation of protrusions, that have been well described at the level of individual cells. Here, we chose to focus on its role in epithelial tissue, which is rising attention in recent works. We discuss how the cellular activities of the Arp2/3 complex drive epithelial dynamics and/or tissue morphogenesis. In the first part, we examined how this complex influences cell-cell cooperation at local scale in processes such as cell-cell fusion or cell corpses engulfment. In the second part, we summarized recent papers dealing with the impact of the Arp2/3 complex at larger scale, focusing on different morphogenetic events, including cell intercalation, epithelial tissue closure and epithelial folding. Altogether, this review highlights the central role of Arp2/3 in a diversity of epithelial tissue reorganization.

## Introduction

Epithelia are sheets of tightly connected epithelial cells that cover structures and organs. They are very dynamic, need to be regularly renewed for correct tissue homeostasis and can undergo important reorganization including epithelial migration, sealing, remodeling. Understanding how epithelia orchestrate these different processes constitutes an important question in the field.

The actomyosin network plays an essential role in epithelial dynamics by generating forces in individual cells that can then be transmitted to the tissue thanks to cell-cell junctions or cell-matrix adhesions (Mao and Baum, 2015). Among the regulators of the actomyosin cytoskeleton, the actin-related protein 2/3 (Arp2/3) complex promotes the nucleation of branched actin filaments when activated by nucleation promoting factors (NPFs) such as WASp and WAVE complex (Higgs and Pollard, 2001), themselves under the respective control of Rho GTPases Cdc-42 and Rac1 (Pollitt and Insall, 2009). While the Arp2/3 complex has been extensively studied in cell culture (for review see (Rotty et al., 2013)), there is now a growing interest in studying its function in epithelial tissue dynamics in multicellular organisms (Lechler, 2014). In epithelial tissue, Arp2/3 and NPFs promote the formation and the maintenance of both adherens and tight junctions (Bernadskaya et al., 2011; Herszterg et al., 2013; Zhou et al., 2013; Sasidharan et al., 2018; Chánez-Paredes et al., 2021) as well as their turnover, through the role of Arp2/3 in endocytosis (Georgiou et al., 2008; Leibfried et al., 2008; Sasidharan et al., 2018), thus conferring to Arp2/3 an important role in the formation and the integrity of the epithelial barrier (Bernadskaya et al., 2011; Zhou et al., 2013; Chánez-Paredes et al., 2021). Moreover, loss of function of Arp2/3 also impairs vesicle trafficking (Patel and Soto, 2013) and, in mammalian small intestine, transcytosis and lipid absorption, resulting in the death of newborn mice (Zhou et al., 2015). In addition, Arp2/3 is involved in collective epithelial cell migration through the formation of focal adhesion formation and polarized protrusions in the direction of migration to ensure, for example, gut epithelial turnover (Krndija et al., 2019), repairing damage to epithelial barrier (Li et al., 2021), or morphogenesis (Olson and Nechiporuk, 2021). At the level of individual cells, the Arp2/3 complex has been shown to possess multiple cell-autonomous functions in cell adhesion, vesicle trafficking and migration. However, the Arp2/3 complex is also involved in larger scale events allowing different cells to communicate and cooperate.

In this review, we choose to focus on the role of the Arp2/3 complex in the modulation of the cell environment, going from the communication between neighboring cells to cell cooperation at the tissue level.

## Arp2/3 in Cell-Cell Cooperation

Cells that compose a tissue need to communicate and cooperate to ensure homeostasis and/or proper morphogenesis. Here we describe two examples of cell-cell communication and cooperation in which Arp2/3 is involved: the cell fusion and the apoptotic cell corpse clearance.

### Cell-Cell Fusion

**FIGURE 1:**
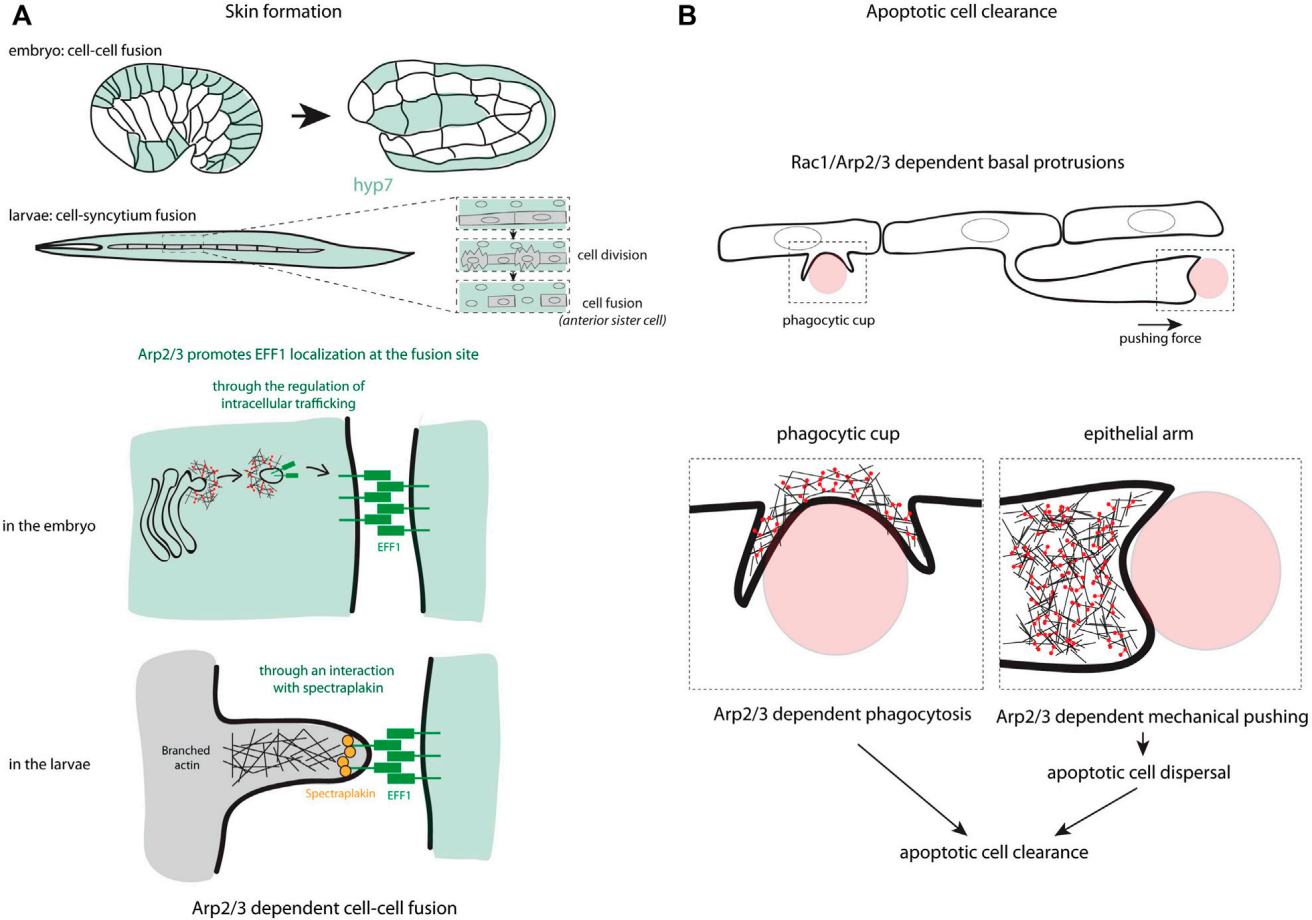
Arp2/3 functions in cell-cell cooperation. **(A)** The different steps in the process of skin formation through cell-cell fusion in *C*. elegans are presented. These schemes highlight the different ways used by the Arp2/3 complex to localize the fusogen EFF-1 at the fusion site, either through the regulation of intracellular trafficking in the embryo or through an interaction of EFF-1 with spectraplakin in larvae. **(B)** Two mechanisms controlled by Arp2/3 used in epithelial sheet to eliminate apoptotic cells are schematized including the formation of phagocytic cup and the formation of an epithelial arm pushing the apoptotic cells to disperse them in the tissue and favor their rapid removal.

The cell-cell fusion is a crucial event in the development and homeostasis of many organisms, where the plasma membrane of adjacent cells merges to generate a syncytium. This mechanism participates, for example, in the formation of muscles, bones, placenta, lens of vertebrate eyes as well as in immune response and fertilization (Pötgens et al., 2002; Chen et al., 2007; Shi et al., 2009). At the molecular level, it has been shown that transmembrane fusogenic proteins are essential for the eukaryotic cell fusion (Avinoam and Podbilewicz, 2011) and studies on muscle development have demonstrated that actin polymerization is also implicated in the mechanism of cell fusion (Kim et al., 2015). An interplay between fusogens and the actin cytoskeleton and its regulators were shown in cell culture (Shilagardi et al., 2013; Sumida and Yamada, 2015). Interestingly, this interplay has also been described in epithelial tissues (Yang et al., 2017; Zhang et al., 2017). In *C. elegans*, two successive fusion processes occur in the embryo and in the larvae, leading finally to the formation of the Hyp7 syncytium. Of note, the fusogen EFF-1 plays an essential role in cell-cell fusion and the depletion of WASp, Arp2/3 complex or both by RNAi lead to a delayed cell-cell fusion in both embryos and larvae (Yang et al., 2017; Zhang et al., 2017). If the WASp-Arp2/3-dependent actin polymerization promotes cell fusion at both stages, it influences the recruitment of EFF-1 differently, may be due to the distinct nature of the fusion processes (cell-cell versus cell-syncytium fusion, Figure 1A). In larvae, the actin polymerization at the plasma membrane recruits and stabilizes EFF-1 to fusion sites through the direct binding to spectraplakin (Yang et al., 2017). In contrast in embryos, the actin polymerization controls the localization of EFF-1 through its transport by intracellular vesicles, and its transient localization at the plasma membrane to promote cell fusion (Zhang et al., 2017). In both conditions, WAVE does not seem involved since its repression does not perturb the cell fusion (Patel et al., 2008; Yang et al., 2017; Zhang et al., 2017).

The cell-cell fusion also contributes to the epithelial wound closure by generating polyploid cells through the Rac signaling pathway, as shown in *Drosophila* (Losick et al., 2013). It could be interesting to evaluate the role of the Arp2/3 complex in the context of polyploidization-dependent re-epithelization. Moreover, after an epithelial injury, a rapid polymerization of actin is observed at the wound site, which in turn leads to the recruitment and the accumulation of the fusogenic protein EFF-1 to provide an efficient tissue repair (Meng et al., 2020). These data suggest a crosstalk between cell fusion machinery and actin polymerization during tissue sealing that could be interesting to investigate further.

### Apoptotic Cell Corpse Engulfment

Clearance of apoptotic cells is an important step in development and tissue homeostasis to avoid inflammation and auto-immune responses due to necrosis of apoptotic cells. While this process is mainly achieved by professional phagocytes, epithelial cells can also have a phagocytosis function.

In *C. elegans*, hypodermal cells hyp7 (and its precursors in embryo) have this capacity. After recognition of the “eat me” signal at the surface of apoptotic cells or midbodies, hypodermal cells activate signaling pathways to internalize cell corpses. The major hallmark of this internalization is the formation of an actin halo around cell corpses or midbodies (Chai et al., 2012; Li et al., 2012), which resembles the formation of the phagocytic cup (Botelho and Grinstein, 2011). Interestingly, Wu and his colleagues have shown that WASp and the Arp2/3 complex participates in the formation of the phagocytic cup through a Rac-dependent signaling and are required for an efficient apoptotic cell corpse engulfment (Wu et al., 2017). Consistent with these previous data, the Arp2/3 complex is also essential in the apoptotic cell clearance in zebrafish and mouse embryos (Hoijman et al., 2021). Interestingly, in addition to the formation of the phagocytic cup, the Arp2/3 complex also participates in the generation of epithelial arms at the basal part of the epithelium (Figure 1B). These arms push apoptotic cells, which results in the dispersion of apoptotic corpses and improves the overall clearance efficiency by the surrounding epithelial cells (Hoijman et al., 2021). This highlights the major role of the branched actin regulators in epithelial cell cooperation for the elimination of cell corpses.

## Arp2/3 During Morphogenetic Events

The Arp2/3 complex can also modulate the cell environment at a larger scale. To illustrate this point, we describe here how the branched F-actin network and its regulation influence morphogenetic events going from epithelial closure to cell intercalation and tissue folding.

### Developmental Tissue Enclosure and Epithelial Repair

Epithelium sealing is a developmental morphogenetic process corresponding to a gap closure mechanism. Given the high degree of similarities with the repair mechanism of damaged tissue, epithelium sealing is considered as a good model of wound healing (Wood et al., 2002; Martin and Parkhurst, 2004); allowing to dissect the cellular and molecular actors involved in an integrated system. Two different ways have been described to seal an epithelial gap; either cell crawling through the generation of large actin-rich protrusions, or a purse-string process, through assembly of a supracellular actomyosin contractile cable (reviewed by (Begnaud et al., 2016)). We discuss the role of Arp2/3 in both mechanisms, in different examples including the *C. elegans* ventral enclosure, the *Drosophila* dorsal closure and wound healing, studied in both *Drosophila* and epithelial monolayers.

**FIGURE 2:**
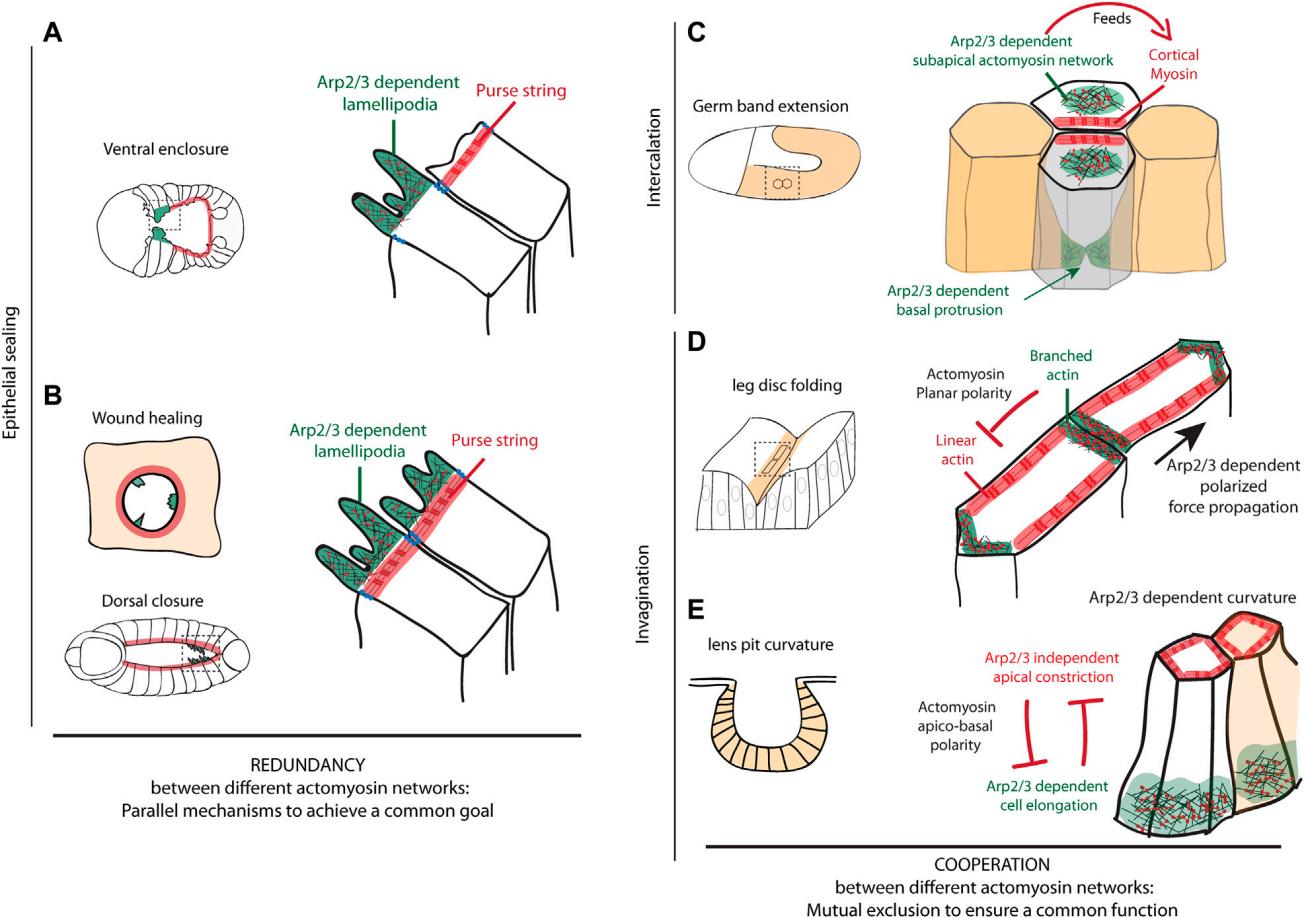
Arp2/3 functions during morphogenetic events **(A–B)** Epithelial sealing mechanisms are schematized, including ventral enclosure in *C. elegans* **(A)**, epithelial wound healing and dorsal closure in *Drosophila* **(B)**. These schemes show that Arp2/3 plays an important role during epithelial sealing in the formation of lamellipodia. They further highlight that branched and linear actin networks act in parallel in these different model systems to close a gap separating two epithelial sheets, thus showing a functional redundancy **(C–E)** Schemes of cell intercalation mechanism during germ band extension in *Drosophila* **(C)** and tissue invagination mechanisms in *Drosophila* leg disc and during lens pit formation in mouse **(D–E)** are presented. They summarize the different subcellular localizations of branched actin network Arp2/3-dependent during these remodeling events. These branched networks can either **(C)** form a subapical meshwork excluded from the cortex feeding the cortical accumulation of myosin, **(D)** be planar polarized and located preferentially in a subset of junctions while linear actin show a complementary localization favoring the polarized transmission of forces across the tissue and ensuring fold robustness or **(E)** form a basal network required for cell elongation while being excluded from the constricting apex and contribute to tissue curvature. These examples further show that branched and linear actin networks can cooperate, while showing a mutual exclusion spatially, to ensure a common function such as tissue elongation, polarized force propagation or tissue curvature.

The *C. elegans* embryo ventral enclosure relies on the collective migration of anterior epidermal leading cells towards the ventral midline where they create new junctions with their contralateral cells (Chisholm and Hardin, 2005). In the first step of this process, cells migrate using large actin-rich protrusions, which depend on actin polymerisation. This has been shown by pharmacological inhibition experiments with cytochalasin D (Williams-Masson et al., 1997) and inhibition of the Arp2/3 complex, WASp or WAVE (Sawa et al., 2003; Patel et al., 2008), which prevents protrusion formation and epidermal closure. This function is cell autonomous as shown by the expression of Arp2/3 complex and WASp specifically in epidermal cells. Furthermore, these proteins colocalize at the leading edge of migrating cells reinforcing the potential involvement of Arp2/3-WASp in actin polymerization during ventral enclosure (Sawa et al., 2003). Supporting this role, the upstream regulator of WASp and Arp2/3, the RhoGTPase CDC-42, together with a RhoGAP - RGA-7 -, has been shown to control actin-rich lamellipodia formation at the leading edge of the leading cells (Ouellette et al., 2016). The formation and the dynamics of lamellipodia, as well as ventral enclosure efficiency, also rely on Ena/VASP and WAVE binding (Havrylenko et al., 2015). Thus, a branched actin network, controlled by Arp2/3, WASp, WAVE, CDC42 and RGA-7, is required at the leading edge for the formation of dynamic lamellipodia and to ensure ventral enclosure in *C. elegans*. Of note, in a second step, a purse string mechanism is taking place, based on actin microfilaments, and has been proposed to pull together the edges of the hypodermal sheet at the ventral midline (Williams-Masson et al., 1997) (Figure 2A). These two mechanisms, spatially restricted to different subgroups of cells, appear to be complementary in ventral enclosure.

This complementarity has also been observed during wound healing, where Arp2/3 regulates the formation of lamellipodia at the leading edge and a supracellular cable of actin bundles generates an Arp2/3-independent purse-string mechanism, as shown *in vivo* in *Drosophila* notum (Antunes et al., 2013; Begnaud et al., 2016). If these two mechanisms are often exclusive, they can also coexist and interplay (Begnaud et al., 2016). However, the role of Arp2/3 is rarely assessed. Interestingly, in epithelial monolayer *in vitro*, it has been shown that the repression of Arp2/3 by CK666 - a pharmacological inhibitor of Arp2/3 - induces a switch towards a purse-string mechanism-dependent repair, indicating that these two mechanisms are complementary and redundant to ensure wound healing (Ajeti et al., 2019). This complementarity could also be at play during *Drosophila* dorsal closure. During this process, actin-polymerization-dependent filopodia occurs in parallel to the formation of a supracellular actomyosin cable-dependent purse-string mechanism (Jacinto et al., 2002; Dobramysl et al., 2021) that has been shown to be dispensable to ensure dorsal closure (Ducuing and Vincent, 2016) (Figure 2B).

Thus, while Arp2/3 complex is an essential contributor of the gap closure by cell crawling through the generation of actin-rich protrusion, its activity seems totally dispensable for purse-string-dependent closure. In some context, a switch from one mechanism to the other can occur to ensure the robustness of gap closure events (Ajeti et al., 2019). Whether this is a conserved mechanism remains an open question.

### Cell Intercalation

Another collective mechanism involving Arp2/3 is cell intercalation, a morphogenetic process used to increase the global length of a tissue without changing the number of cells. This process of cell intercalation can be regulated by different cellular programs, through the control of either their apical junctions or their basal protrusive activity (Walck-Shannon and Hardin, 2014). Here, we summarize how the branched actin network can influence this process through two examples.

In the *Drosophila* embryo, cell intercalation is a key process of germ-band extension (Bertet et al., 2004). This process is associated with the shrinkage of dorso-ventral junctions (perpendicular to the elongation axis), that is driven by the polarized activity of the actomyosin cytoskeleton at the junction (cortical location) (Bertet et al., 2004). The cortical accumulation of non-muscular myosin II (hereafter called myosin) is fed by the medial pool of myosin, displaying anisotropic flow of myosin (Rauzi et al., 2010). This medial pool of myosin depends on the regulation of branched actin polymerization by WASp. Indeed, while WASp is normally downregulated at the cortex by the JAK/STAT pathway, a constitutively activated form of WASp located at the membrane is sufficient to prevent actomyosin cortical accumulation and favor the formation of a medio-apical meshwork. Altogether, this suggests that WASp and presumably Arp2/3 need to be excluded from the cortex and are necessary for the formation of a medio-apical meshwork of actomyosin that feeds DV junctions through the formation of a myosin anisotropic flow (Bertet et al., 2009). Finally, this accumulation of cortical myosin in disassembling junctions leads to junction shortening, the formation of a rosette and the creation of new interactions between dorsal and ventral cells. Preceding this apical rearrangement, polarized basolateral protrusions are formed in dorsal and ventral cells. These protrusions are dependent on Rac1 activity (Sun et al., 2017), which indicates that actin polymerization occurs basally. However, whether this depends on the basal activity of the Arp2/3 complex remains unknown.

The formation of basal protrusion is also observed during cell intercalation in *C. elegans* embryo. In this model system, two rows of dorsal epidermal cells intercalate to form a single row along the dorsal midline. To insert between each other, cells extend polarized basal protrusions oriented in the direction of the rearrangement (Chisholm and Hardin, 2005). It has been demonstrated by Walck-Shannon and her colleagues that WAVE and WASp act redundantly, under the respective regulation of Rac (ced-10) and RhoG (mig-2), to generate these protrusions (Walck-Shannon et al., 2015). Although not shown, these data also highly suggest that this could occur through the activity of the Arp2/3 complex.

The cell intercalation therefore seems to depend on the activity of the Arp2/3 complex and its regulators either directly, by generating basal protrusions at the leading edge of the cells, or indirectly, acting on the cortical myosin enrichment from a medial location (Figure 2C).

### Tissue Folding

Epithelial invagination is a key process of embryonic development and organ shaping allowing a 2-dimensional epithelial sheet to acquire a 3D architecture. Many processes have been described in the litterature to perform epithelial bending (Pearl et al., 2017) but only few studies involve Arp2/3 complex in this morphogenetic process. Here, we will describe two mechanisms driven by this actin nucleator.

Studying the development of both mammalian salivary gland and teeth, Li and colleagues showed that the epithelial bending, occurring during the morphogenesis of these organs, was driven by a ‘vertical telescoping’ of cells engaged in this process (Li et al., 2020). Similar to the unfolding of a collapsible cane, this mechanism is biologically explained by the vertical migration of external cells of the epithelium that leads to a depression of more internal cells. Moreover, the authors demonstrated that this mechanism occurs without cell shape changes but requires the generation of centripetally polarized apical protrusions. Indeed, when they blocked the protrusion formation either widely, using cytochalasin D, or more specifically, using an inhibitor of the Arp2/3 complex, they observed no more invagination, suggesting that the Arp2/3 complex is an essential factor of both salivary gland and tooth morphogenesis.

In our recent work, the Arp2/3 complex has been also involved in epithelial folding robustness, through a novel role on force channeling (Martin et al., 2021). Indeed, using the *Drosophila* leg precursor epithelium, whose morphogenesis is well stereotyped with the formation of four strictly parallel folds, we have shown that the depletion of subunits of the Arp2/3 complex induced misoriented folds, while the developmental pattern was correctly located. This phenotype, which exhibits a high variability from one individual to another, is characteristic of a lack of morphogenesis robustness. Furthermore, we showed that this variability was associated with a loss of the actomyosin planar polarity at the level of adherens junctions together with the resulting anisotropic tension. Our data also revealed that the Arp2/3 complex participates in the establishment of the actomyosin planar polarity to avoid forces scattering and bias propagation of forces in the fold axis (Figure 2D). Finally, all of these characteristics confer a mechanical robustness to the tissue against random mechanical perturbations that would otherwise disrupt the reproducibility of the final shape of the epithelium. However, how the Arp2/3 complex regulates the anisotropic distribution of the actomyosin network at the molecular level remains unknown.

Another illustration of the role of the Arp2/3 complex in epithelial invagination, is the formation of the developing mouse lens (Chauhan et al., 2011). Interestingly, in this tissue, RhoA and Rac1 lens mutants generate opposite invagination phenotypes in terms of lens curvature. On the one hand, RhoA mutants exhibit reduced apical constriction and increased cell elongation, which results in a lens pit with a more open shape. On the other hand, in Rac1 mutants, cells are more constricted apically and less elongated apico-basally, which results in a closer lens pit. Using phospho-myosin as a marker of RhoA activity and the intensity of an Arp2/3 component as a marker of Rac1 activity, the authors further show that RhoA activity is increased in Rac1 mutant, while Rac1 activity increases in RhoA mutant both apically and basally. These results reveal that a mutual antagonism between RhoA dependent apical constriction and Rac1 dependent cell elongation is at play to control cell shape in the lens pit, and as a consequence to control the shape of the placode (Figure 2E).

Altogether, these data indicate that Arp2/3 complex is an important regulator of epithelial invagination, either through a well-known role in the formation of actin-rich protrusion or through a newly identified function in force channeling.

## Conclusion

Arp2/3-regulated branched actin network is known to be involved in diverse cellular processes, including the formation of lamellipodia and filopodia, endocytosis and/or phagocytosis and cell-cell adhesion dynamics. Here, we chose to focus particularly on the function of Arp2/3 in epithelial dynamics, from cell-cell interaction to tissue scale reorganization.

In the first part of this review, we describe the function of Arp2/3 in cell-cell fusion and apoptotic cell clearance. Interestingly, these studies show that Arp2/3 can play an important function in targeting molecules or cell bodies at specific locations. It is the case of the fusogen protein EFF1, which accumulates in the region of cell-cell fusion in an Arp2/3 dependent manner. It is also the case for apoptotic bodies, which are dispersed within an epithelial sheet by an Arp2/3-dependent cellular extension to favor apoptotic cell clearance (see Figure 1).

In a second part, we focus on Arp2/3 impact at a broader scale in tissue morphogenesis including its function in epithelial sealing, cell intercalation and tissue folding. These studies reveal that branched and linear actin networks can work in parallel to close the gap existing between two epithelial sheets, but can also show mutual exclusion spatially while cooperating functionally to favor tissue elongation, cell shape changes or to favor polarized force propagation (see Figure 2). Overall, this review recapitulates different scenarios highlighting the different types of cooperation existing between different actomyosin networks.
